# Supplementary material for: Predation and fragmentation portrayed in the statistical structure of prey time series
Source: BMC Ecol. 2009 May 6;9:10. doi: 10.1186/1472-6785-9-10 (PMC2689204; doi:10.1186/1472-6785-9-10)
Supplement: Additional file 2 — Voles and related classes ODDox Documentation. ODDox documentation of the agent-based model (ALMaSS) applied by Hendrichsen et al. The documentation is started by activating main.html. [file 1472-6785-9-10-S2.zip › Vole_ODDox/functions_func_0x6f.html]

ALMaSS ODDox: Class Members - Functions

- Main Page
- Related Pages
- Classes
- Files

- Alphabetical List
- Class List
- Class Hierarchy
- Class Members

- All
- Functions
- Variables

- a
- b
- c
- d
- e
- f
- g
- h
- i
- k
- l
- m
- n
- o
- p
- r
- s
- t
- u
- v
- w
- ~

### - o -

- OnArrayBoundsError()
  : TALMaSSObject- OnFarmEvent()
    : TAnimal
    , Vole\_Female
    , Vole\_Male- OnInfanticideAttempt()
      : Vole\_Female- OnKilled()
        : Vole\_Female
        , Vole\_Base
        , Vole\_Male- OpenFile()
          : probe\_data- OpenTheBreedingPairsProbe()
            : Population\_Manager- OpenTheBreedingSuccessProbe()
              : Population\_Manager- OpenTheCIPEGridOutputProbe()
                : Population\_Manager- OpenTheFledgelingProbe()
                  : Population\_Manager- OpenTheMonthlyRipleysOutputProbe()
                    : Population\_Manager- OpenTheReallyBigProbe()
                      : Population\_Manager- OpenTheRipleysOutputProbe()
                        : Population\_Manager- operator()()
                          : CompareX
                          , CompareY
                          , CompareState- OrganicCattle()
                            : OrganicCattle- OrganicPig()
                              : OrganicPig- OrganicPlant()
                                : OrganicPlant- OverlapMyTerritory()
                                  : TPredator- Owl()
                                    : Owl

---

Generated on Thu Jan 22 14:13:45 2009 for ALMaSS ODDox by 
 1.5.6
